# Communication as a Key Performance Indicator in Employer Branding in the Context of the Social Economy—A Quantitative Study

**DOI:** 10.3390/bs14040303

**Published:** 2024-04-07

**Authors:** Michael P. Heide, Silvana Prodan, George Lazaroiu, Barbara Kreis-Engelhardt, Alexandru-Mihai Ghigiu

**Affiliations:** 1 Department of Marketing, Faculty of Economics and Business Administration, Babeş-Bolyai University, 400591 Cluj-Napoca, Romania; silvana.prodan@econ.ubbcluj.ro; 2 Faculty of Business and Law (FWR), Nürtingen-Geislingen University of Applied Sciences (HfWU), 72622 Nürtingen, Germany; barbara.kreis-engelhardt@hfwu.de; 3 Department of Languages and Communication, Inter-Languages Center: Text, Image, Language (TIL) (EA4182), University of Burgundy, 21000 Dijon, France; 4 Faculty of Science and Engineering, Curtin University, Bentley, WA 6102, Australia; george.lazaroiu@spiruharet.ro; 5 The Intelligent Communications and Computing Lab, Toronto Metropolitan University, Toronto, ON M5B 1G3, Canada; 6 Department of Economic Sciences, Spiru Haret University, 030045 Bucharest, Romania; 7 Department of International Relations and European Integration, National University of Political Studies and Public Administration, 012244 Bucharest, Romania; mihai.ghigiu@dri.snspa.ro

**Keywords:** employer branding, communication, signal theory, employee survey, structural equation model, social economy

## Abstract

Performance measurement refers to the systematic evaluation and analysis of the performance and results of business processes, initiatives, or strategies. This study discusses the crucial role of communication using signaling theory in employer branding in the context of the social economy organization (SEO). The aim is to measure employee satisfaction in concrete terms and to determine the status quo of the communication culture of the organization under investigation in order to develop an employer branding strategy based on the results. The authors use an employee survey as a quantitative research method and limit the data collection to the EU member state of Germany considering the research background. The results provide insights into the specific communication policy in relation to employer branding. The focus here is on (digital) communication. Organizations need to understand how communication strategies directly influence the perception of the employer brand in the social economy. Furthermore, practical implications are derived in order to increase employer attractiveness. Concrete recommendations of action for SEOs should help them be successful in the competition for qualified specialists and talent.

## 1. Introduction

The world is changing. Companies are facing challenges in the provision of social services. The digital transformation has already begun and affects all sectors and fields of work [1]. The focus of the digitalization strategy is on the virtualization and networking of the real world as well as the platform-based management of value chains. Social economy organizations (SEOs) provide social, medical, or care services in a stakeholder-oriented manner. Managing scarce resources and ensuring the competitiveness of companies both today and in the future is of great importance to ensure the provision of care [2]. The resulting structural changes require customized, holistic concepts that need to be developed and implemented [3]. Bottlenecks in the labor market hinder the necessary process steps and show companies the relevance of signals to mitigate this problem. In this context, signaling theory offers useful suggestions for describing the behavior of two parties, considering existing information asymmetries. The parties here can be individuals or organizations. The sender decides how and what information to transmit to the receiver and the latter decides how to interpret the signals/information [4,5].

Signal theory [6] was selected from the literature as the theoretical frame of reference for this study. On this basis, the following research questions were formulated:

1. How can social economy organizations in Germany improve their employer image based on a signal-theoretical communication model?
2. What role does communication culture play in the interplay among the working atmosphere, training, and employer image?

For this purpose, 129 questionnaires with 12 subject areas were distributed to employees at the main site of SEO Häussler Ulm from October to November 2023. In addition, the authors examined the presence and effect of signals in relation to the topic areas (constructs).

The originality of this project lies in the fact that the quantitative data collection by means of a questionnaire was adapted to the needs of social economy organizations. To support the development of an employer branding strategy based on continuous improvement in the employer image, the aim was to determine the status quo of the communication culture in the organization studied. The questionnaire developed can also be applied to other SEOs with similar operational structures. The structure of this article is as follows: The first section provides an overview of the literature and presents the research questions. The third section deals with the research method leading to the (empirical) results and discussion before conclusions and research limitations are presented.

## 2. Theoretical Framework

Signal theory is used in social science research to describe the dynamics of communication between people and organizations and to make explanatory statements about the effect of transported information and any existing information asymmetries [4,5]. Information has a considerable influence on an individual’s decision-making processes. Knowledge of the structure of effects of a communicative information flow improves the understanding of the decision-making of actors [7] in communicative interaction [4]. In social relationships, the perception of signals by actors is a prerequisite for their effectiveness. Signaling theory has become increasingly important, especially in times of skills shortages. In addition to the areas of strategic management and corporate management, this is particularly true in the field of recruitment research [8].

Job applicants use targeted signals to give companies indications of their performance potential. Companies use signals to present themselves as an attractive employer to potential applicants [9]. Signals help applicants determine how well their needs match the organizational environment. The information circulated is usually positive [10,11]. Signaling creates a framework for how external corporate communication can influence the perception of employer attractiveness among potential applicants [8]. The communication process generates both an internal and an external effect. Internal communication strategies address the groups of people who represent the company to the outside world, while external communication strategies focus on the stakeholder groups that should become aware of the company [8].

The three main elements of signaling theory are the signal, the signal generator, and the receiver. Signalers typically present a variety of details, including information about products and services, news of preliminary sales results, and details about other aspects of the company that are helpful to outsiders. According to signaling theory, when outsiders receive this information, they are called recipients of the message [5,12]. For example, employees and future employees are recipients of signals about job advertisements. A receiver’s attention span and how intensively receivers search for signals in their environment are two factors that influence the success of the signaling process. The importance increases even further with weak signals that receivers can miss if they are not looking for them. Actors with information advantage (signalers) can send signals that contain reliable information about normally undetectable aspects to prevent ineffective outcomes. Receivers who do not have the data in question can absorb and evaluate the signals and make smart decisions [5,12]. A job applicant knows more about his or her portfolio on the job market than the company. Companies may be suspicious of applicants’ social media accounts because they have an interest in sharing positive information. This makes it difficult for employers to find qualified applicants [12]. Signaling theory is used in business contexts where parties with different levels of knowledge work together. The focus of the use of signaling theory is primarily on entrepreneurship [12,13]. Colombo [13] provides an in-depth summary of which new venture signals work best for equity investors, company angels, and crowdfunders based on a selection of 68 articles, while other authors provide [4] an overview of some signaling studies from management research. Bafera and Kleinert [12] conduct a systematic literature review of 172 studies on entrepreneurship and provide a taxonomy of 18 signaling elements that can serve as a useful structure for future research on signaling theory. Studies in signaling theory have examined the signaling that occurs throughout the hiring process [14]. Using signaling theory, other authors [15] review the development of HR skills research and examine the signals employers send when hiring HR professionals in New Zealand. Since most studies focus on management and entrepreneurship, the originality of this research rests on the authors’ application of signaling theory to talent management and HRM in the context of social economy organizations based in Germany.

### 2.1. Employer Branding Strategies

The main objective of marketing is to inform consumers about a brand, product, or service. A successful marketing strategy requires the existence of a relationship between the marketer and the customer. In today’s business world, customer satisfaction is an essential factor. The idea of delighting customers through service excellence is an essential part of service research and practice [16,17]. The customer journey is defined as a series of touchpoints that customers experience during their decision-making process [18].

Customers no longer primarily buy goods and services, they buy brands. This indicates that the brand experience has a significant influence on customer loyalty. The perception of a brand is sustainably strengthened by positive experiences [19]. Consequently, investment in marketing is crucial, and it is imperative for companies to consistently deliver unique experiences to their customers. Integrating social awareness into marketing techniques is crucial, especially for SEOs [20]. Through communication initiatives to build social awareness and deepen the relationship between the employer brand and desired social attributes, organizations plan and manage their job offerings with consistent application of marketing principles [8,21]. Potential employees often visit the social media channels of their respective companies. Thus, it is necessary to send signals that enable potential applicants to assess the character of the company as a potential employer [9]. Companies generally use various social media platforms and aim to positively influence the perception of their employer brand through informative, interactive, and entertaining activities. By communicating this as part of an employer branding strategy, job seekers should be made aware of the company and, if suitably qualified, be hired [22]. The perceived attractiveness and reputation of the company act as mediating factors for the most positive association possible, the content published on social media platforms, and, at the same time, the perception of the company as an attractive employer [23]. It is important to develop an employer branding strategy to meet the expectations and needs of target groups such as Generation Z. It is an important building block for countering the consequences of the shortage of skilled workers [8].

#### 2.1.1. The Use of Social Media as Part of Branding Strategies

Social media and company websites enable a rapid exchange of information. This can build a strong bond between employees and companies [24]. Social media managers are responsible for the online presence of companies on social media. They take responsibility for communicating the company’s brands and thus also for employer branding [25,26]. Websites and social media can be used specifically as important platforms for communicating values and employer qualities. The availability of information about a company on social media has a positive effect on the perception of the company’s image. This in turn can be linked to employer attractiveness [27,28].

Key performance indicators (KPIs) are used to measure the success of branding strategies and to evaluate performance at different organizational levels [29,30]. KPIs are quantifiable measures designed to help track progress toward organizational goals, evaluate performance, and support strategic decisions [29]. KPIs can be applied in different areas of an organization, including finance, marketing, human resources, and even employer branding, to assess and improve the success of initiatives. In terms of employer branding in the social economy, communication as a KPI can include various aspects aimed at measuring the effectiveness of communication strategies to build and maintain the employer brand [31,32] such as the following:

- Online presence: The effectiveness of digital communication strategies can be measured by the number of followers, likes, shares, and comments on social media platforms as well as the reach of content.
- Reputation as an employer: A company’s reputation as an employer can be assessed through surveys (MAB) and reviews on platforms such as kununu.com.

The use of information and communication technologies (ICTs) is indispensable because of the changed framework conditions in the HR industry [33,34]. In the age of global connectivity, innovation is a crucial component of competitiveness. The literature shows [35] that digital technologies and infrastructures are significantly changing the collaborative approach of companies. Platforms serve as an acquisition channel and source of information [26,36,37].

#### 2.1.2. The Use of Artificial Intelligence as Part of the Branding Strategy

Artificial intelligence (AI) is increasingly being applied to social media [38] and used in HR to find excellent candidates even before they apply for a job. The goal of the candidate acquisition phase of the talent lifecycle is to find as many people as possible with the required skills for a specific position. If a candidate is a good fit and can deliver the required performance [39], s/he is encouraged to apply for a job. Using data about the candidate collected during the application process, recruiters can use AI to accurately predict a candidate’s future performance and assess whether his/her profile matches the job requirement [40].

AI technologies, such as deep learning or search and chat solutions [41], can have the following effects: higher acceptance through satisfaction with the technology, better professional communication, and improved clinical practice and care through better history taking. In emergency medical situations, technology is a useful decision-making tool that can improve clinical practice and reduce workload [42]. The development of modern ICTs has enabled smooth integration of the various providers involved in patient care. The refocusing of services to the community and proximity to the patient in their local unit can be facilitated by such technologies. ICTs integrated with computers, smartphones, sensors, and web-based applications are used by digital health systems to facilitate the efficient delivery of healthcare services and information [43]. Experts from the fields of health sciences, software engineering, communication, and law as well as related disciplines are involved. In the scientific debate, it is important to consider the impact of technology use on human behavior [44].

Social media supports interprofessional communication and facilitates onboarding and, at the same time, sustainable learning in the workplace. These technologies enrich everyday working life. They improve the mental health of health and social care professionals, their motivation, and their sense of self-determination [42].

### 2.2. Talent Management

To be successful in today’s increasingly complex global market, companies need employees with high potential and, at the same time, with experience [2,45]. Managers responsible for HRM must recruit the best workers and ensure their organizational retention [45]. The person-related combination of ability, experience, and skill is described as human capital. Therefore, it is crucial to understand how to manage outstanding employees, how to invest in them, and, at the same time, how to increase the productivity of the organization [46]. Many companies know that the best HRM techniques are closely linked to the company’s strategy and culture [47]. Nevertheless, talent management lacks certain framework conditions [46,48]. For example, the career development program tailored to the respective person can be mentioned as an organizational competitive advantage [49,50]. Because of a lack of skilled workers and the associated challenges of retaining skilled workers in the organization in the long term, professional talent management in the healthcare sector is of central importance to ensure efficient patient care [51].

To keep pace with rapid technological advances and the rise of new generations of workers, including Generation Y and Generation Z, whose job expectations are evolving, companies must implement holistic talent management strategies. In addition, organizations must increasingly integrate people from other cultures and nations. To manage talent effectively, the leadership qualities of those responsible must be continuously evaluated and improved because developing the potential of employees is the primary goal of professional talent management. Companies that support the talent development of their employees create an innovative environment in which expertise, knowledge, and leadership skills can grow. In addition, companies can create productive teams, ensure long-term stability, and achieve qualitative success [52]. The sustainable talent management system is a corporate function that aims to attract, retain, and develop the best employees that the company needs [53,54].

### 2.3. Working Atmosphere, Training, and Image

The productivity of employees depends on how well they perform their assigned tasks, which, in turn, is influenced by their working environment. People spend a lot of time at work. In the context of change, the working environment must always be the focus of top management. This shapes the attitudes and behavior of employees. Changes in the working environment therefore also bring about changes in attitudes and behavior. The management style, the agility of the organizational structure, and the job description are important elements that shape the work environment [55]. However, this is influenced by physical elements such as temperature, ergonomics, and technology. They not only affect the work environment but also have a direct impact on the individual workplace, as well as on work procedures and processes. The working environment and workplace therefore form a unit which, together with behavioral and physical elements such as communication, work–life balance, team spirit, training, development, etc., make up the working atmosphere [56]. This is perceived subjectively by the individual and describes “[…] the quality of social relationships within the organization and the conditions that shape them, how they are perceived by the workforce and how they shape their behaviour […]” ([57], p. 27).

**Hypothesis** **1 (H1).** *A positive working atmosphere has a positive influence on employer image.*

**Hypothesis** **2 (H2).** *A positive working atmosphere has a positive influence on communication in the social economy organization.*

A positive image based on integrity strengthens customers’ trust in the (employer) brand and positively influences their purchasing decisions. This leads to increased customer growth and a boost in sales [58]. As representatives of the corporate image, employees with direct customer contact are often seen as ambassadors, as they play an active role in the external communication [59] of SEOs. They are a valuable tool for monitoring immediate results in the context of service interactions and strategic initiatives [60]. Customer satisfaction and loyalty are shaped by employees’ understanding of their role within the service process [59,61].

**Hypothesis** **3 (H3).** *Communication has a positive influence on the (employer) image of social economy organizations.*

Structured training and development are part of an attractive working environment and working atmosphere. To increase productivity, it is more important than ever to support and actively promote the physical and mental development of employees and their activities [62]. Further training should help employees to reflect on their views on certain topics [63]. It aims to improve individual skills to meet organizational requirements [64]. An effectively designed training program provides the basis for the performance of tasks and at the same time promotes employee motivation [65]. As a component of modern HRM, training and human resource development help in strengthening an organization’s ability to achieve positive results [66]. Kaslon et al. [67] state that structured learning in the workplace provides professionals with targeted qualifications and at the same time shapes a high-performance work culture. The importance of continuing education is considered a decisive factor for the (economic) success of companies worldwide. Further training is not only understood as the acquisition of specialist knowledge [67] but primarily as re-skilling and upskilling to prepare employees for the future in the world of work. This involves interlinking concepts of further training and personnel development [65,66]. Organizational culture—a pattern of values—is an important component of the working environment, as it promotes a productive and positive working atmosphere. A modern, inspiring organizational culture promotes employee performance and helps the team to achieve the company’s formulated (breakthrough) goal [68].

**Hypothesis** **4 (H4).** *Communication has a positive influence on the company’s commitment to continuing education.*

**Hypothesis** **5 (H5).** *Structured education and training have a positive influence on the image of social economy organizations.*

Puncheva-Michelotti et al. [69] state that highlighting diversity in the workplace, internal stakeholder engagement, and growth prospects increase the attractiveness of an employer brand. As a result, companies often advertise measures that promote employee well-being, such as work–life balance and occupational health management [70]. In addition, creating an environment where employees feel a sense of belonging and have the opportunity to act as brand ambassadors to attract new talent increases the attractiveness of the employer [71]. Actively designed initiatives, a well-maintained website, and an up-to-date social media presence of a company have a positive effect on the employer brand [8,72]. Social media can offer added value in the search for skilled workers [26,73]. By applying AI technologies to social media, potential candidates can be identified at an early stage and their suitability for vacancies assessed [40]. The signals sent via websites and social media are processed by the companies. The internal and external (potential) stakeholders are the recipients of the message.

**Figure 1:**
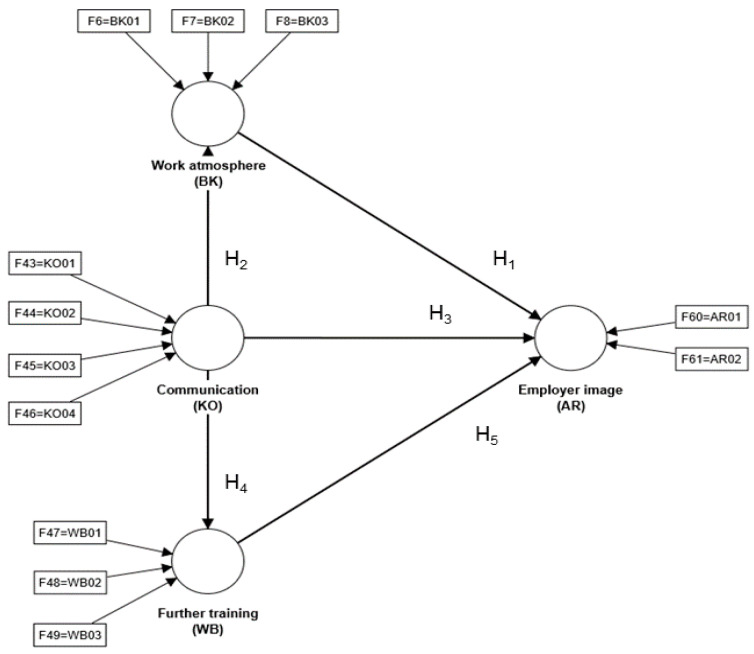
Research model with hypotheses. Source: Own representation.

The constructs mentioned in the research model (see Figure 1) were derived from the literature, which proves their relevance in the context of signaling theory [6]. The sending and receiving of signals regarding communication, working atmosphere, training, and employer image were analyzed, as these facilitate the exchange of information and create connections between companies, employees, future employees, and customers or potential customers [24].

## 3. Materials and Methods

### Research Design

In the context of this article, the choice of model was made in favor of a formative measurement model, as this is a management-oriented study. This measurement model specification enables the identification of drivers (e.g., communication) and thus the derivation of recommendations of action for the SEO [74]. Variance-based estimation using PLS analysis was deployed to test the causal model. As least squares regression is only slightly influenced by small sample sizes, the PLS algorithm is also suitable for small sample sizes. When using PLS analysis, the recommended minimum sample size should be larger than 100 [74,75]. To estimate the minimum sample size, the authors performed a G*Power analysis. The PLS analysis was carried out with software support using SmartPLS version 4.0.9.9 [76]. The content analysis was followed by a quantitative analysis to substantiate the results from the case study [77], an employee survey (MAB) conducted in 2023. The convenience sampling method was used, and the socio-economic organization (SEO) Häussler Ulm was selected. Table 1 below shows a summarized overview of the MAB projects.

In the literature (external benchmark), it is assumed that only a response rate (RLQ) of >70% in the B2C context can be considered representative, which ultimately leads to meaningful survey results [78]. Compared with the MAB 2020 (internal benchmark), the RLQ increased positively by 5 percentage points. The MAB 2023 was used to create a situation analysis of the current corporate culture, which serves as the basis for SEO’s development to optimize service quality for internal and external customers. The paper-based survey was conducted between 30 October and 19 November 2023. From the originally collected data (129 questionnaires), 32 questionnaires with missing data were deleted. Only completely completed questionnaires were considered for further statistical analysis. The results of the G*Power analysis showed that, for three predictors (working atmosphere, communication and further training), an effect size f^2^ ≥ 0.350 would be required with a sample of n = 76 questionnaires. The sample size of n = 97 was therefore appropriate for this study. The age group of the employees was between 30 and 50 years, which corresponds to 46.39%. In general, 83% would recommend the SEO as an employer, whereas only 40% believe that the company’s image on the market is excellent. Table 2 below shows a condensed overview of the socio-demographic characteristics of the sample.

Anonymous, standardized interviews without variation were used to record employee satisfaction at the SEO by means of a questionnaire. The questionnaire was tailored to the needs of the SEO and divided into 14 topic blocks. The questionnaire length for online surveys should be between 20 and 50 questions for the MAB survey type [78]. In addition, the occasion and the topic of the survey determine the questionnaire length. The authors developed a paper–pencil questionnaire with a total of 61 questions (items). Standardized and established measurement instruments were used, such as the (modified) Job Descriptive Index [79]. The Likert scale used with five characteristics is a gradual response scale on which the people surveyed reveal their attitudes.

## 4. Results

The first step is to analyze the correlations between the constructs. Table 3 below shows the correlations.

Furthermore, F2 “My attachment (identification) with this company is very high.” correlates with F60 (r = 0.530), and F2 and F3 “I am very satisfied with my current work situation.” correlates with F61 (r = 0.620).

### 4.1. Quality Check of the Formative Measurement Model

It is not necessary for formative indicators to correlate with each other or to show a strong correlation between a construct and the assigned indicators [80]. In the formative measurement model (see Table 4), the indicators do not represent the same facts, so the methodology of the quality check is limited accordingly [75,81,82]. This article uses the following criteria recommended by the literature [81,83] for the quality assessment of formative constructs: content validity, reliability in terms of indicator relevance, and indicator significance are relevant. The variance inflation factor (VIF) is a recognized indicator for investigating multicollinearity [81,83].

### 4.2. Low Weights

Low weights of individual indicators cannot be interpreted as signs of misspecified measurement models [80,84,85]. Following theoretical and logical considerations, indicators with a low weight are therefore retained in the model in this article. If they are eliminated, this can lead to a distortion of the content of the formative construct, which would result in an incorrect specification of the measurement model [81,82,86].

### 4.3. Variance Inflation Factor (VIF)

As part of the quality assessment of formative measurement models, a problematic level of multicollinearity is assumed from a VIF value ≤ 10, so this guideline value should not be exceeded [81,82,83,84,86]. The calculated values for the variance inflation factors (VIFs) are below the critical threshold for all indicators, meaning that there is no problematic level of multicollinearity (see Table 5). The test for collinearity was carried out by determining the VIF for all items. The highest VIF values were 2.112 ≤ 10 (BK03 and AR02). The analysis of collinearity among the constructs showed that the highest VIF value of the inner model was 2.129 ≤ 10 (WB → AR). The saturated model had an acceptable goodness of fit.

### 4.4. Bootstrapping Procedure

The significance of the weights is also used to evaluate the absolute values of the indicator weights. The indicator significance is determined using bootstrapping based on a t-value. Based on an error probability of 5%, a value of t ≥ 1.960 is set for the significance [81,82,84,86].

### 4.5. Quality Check of the Structural Model

Once a reliable estimation of the constructs by the measurement model has been established, the PLS estimation results of the structural model can be validated. For this purpose, the authors use criteria that allow for an assessment of the path coefficients within the structural model as well as an evaluation of the constructs for their informative and predictive power [75,81]. The coefficient of determination (R^2^) is decisive for the quality check of all endogenous variables of the structural model. The larger the value, the better the regression fits the empirical data. A critical threshold value is not defined in the scientific literature, as this is fundamentally dependent on the respective problem [87].

The R^2^ values for the AR and WB constructs are above the defined reference value and can therefore be classified as moderate—but they are significantly above the value of 0.333. This indicates a moderate predictive power of the structural model (see Table 6 and Table 7). A moderate predictive power can therefore be stated for the model set up in this article.

The intensity, strength, and significance of the impact relationships among latent variables are represented by path coefficients. The values of standardized path coefficients can lie within a value range of −1 to +1. If the values are close to zero, this implies a weak relationship; if the values approach +1 or −1, it can be concluded that the latent variable has a strong influence on its causal successor [80,81,83]. To analyze the path coefficients, Chin [80] defines a minimum value of +0.1 or −0.1 and already sees a significant correlation from a value of ≥+0.2 or −0.2. The significance of the calculated parameters was tested using the t-values determined by bootstrapping. Significance is given if the probability of error is a maximum of 5% (t-value ≥ 1.960) [86]. Significant paths that correspond to the sign defined when forming the hypothesis promote empirical proof of the assumed relationship (see Table 6). Non-significant paths and paths with the opposite sign (compared with the hypothetical assumption) call the corresponding hypotheses into question but can provide important clues to further scientific questions [81,82]. The explanatory contribution of the exogenous variables to the endogenous variable is assessed by the effect size (f^2^). If the value exceeds 0, the existence of a general influence can be assumed.

**Figure 2:**
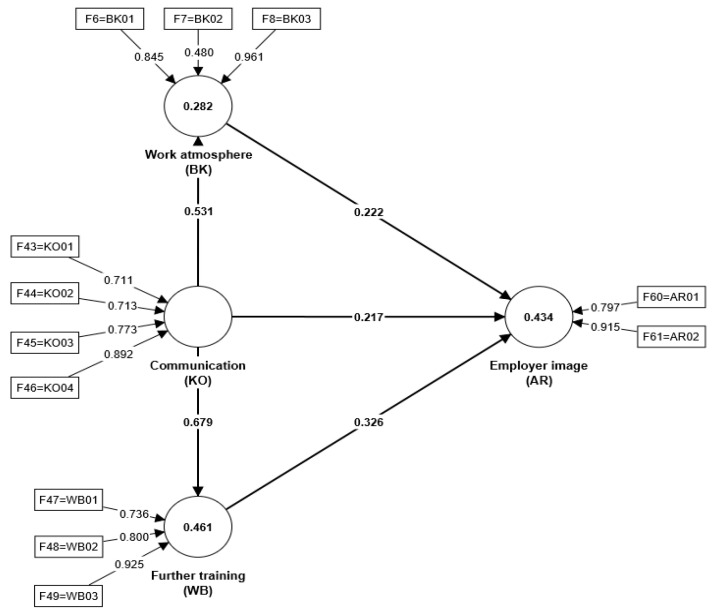
Path model. Source: Own representation.

Figure 2 below shows the calculated path model with the R^2^ values for the constructs BK, AR, and WB, the path coefficients (β), and the respective loadings of the formative indicators.

## 5. Discussion

This section interprets the results of our study and analyzes their significance for the research field. The authors begin with a summary of the most important results before interpreting and discussing the findings. In recent years, employer branding has become an important management topic across all industries. It refers to the way in which an employer brand is designed and communicated to recruit qualified professionals and retain them in the long term.

The PLS analysis performs the estimation in a three-stage procedure and designates the structural model as the inner model and the measurement model of the latent constructs as the outer model. The inner model specifies the relationship between the latent constructs, while the outer model measures the relationship between latent constructs and the correspondingly assigned manifest, directly observable indicator variables [81]. The “inner estimation” determines the weights and construct values of the structural model. The subsequent “outer estimation” determines the weights and construct values of the measurement model. In the case of a formative measurement model, multiple regression coefficients are used to reflect the influence of the indicators on the latent variable [74,86]. As the statistical values of the coefficients of determination (R^2^) show, the R^2^ values are moderate for the AR and WB constructs and weak for the BK construct. Accordingly, the fit of the regression function can only be considered partially good.

Table 8 shows that all path coefficients (β) are greater than +0.2 and, therefore, a positive correlation can be assumed. As can be seen in the presentation of the hypothesis test, three t-values indicate that the path coefficients shown (β) are statistically significant. The effect size (f^2^) assesses the influence of an independent exogenous variable on an endogenous variable. Thus, f^2^ measures the explanatory range of an exogenous variable, for example, KO on the endogenous variable AR. If f^2^ is greater than 0, it can be assumed that the variable has an influence. All effect sizes (f^2^) are greater than 0.02 and mean that effects exist.

H1 suggested that BK positively influences AR. The results obtained (β = 0.222; t-value = 1.647; *p* > 0.05; f^2^ = 0.054) show that the intensity is low. Based on theoretical and logical considerations, H1 is not rejected, but the identified limitation must be considered in the interpretation.

H2 suggested that KO increases BK. The findings obtained (β = 0.531; t-value = 7.506; *p* < 0.05; f^2^ = 0.394) indicate a medium intensity. Previous studies have shown that the work environment requires clear communication guidelines and, at the same time, continuously improves the communication process in the company [3,55,56]. The literature [55] shows that the individual perception of the working environment has a significant influence on employee productivity. The working atmosphere is embedded in the working environment and is shaped by the communication behavior of the actors [56]. Therefore, H2 is confirmed.

H3 suggested that KO positively influences AR. However, the results (β = 0.217; t-value = 1.780; *p* > 0.05; f^2^ = 0.042) show that the intensity is low. Previous studies have focused on entrepreneurial success, which is strongly influenced by (employer) image. As employees with customer contact play a prominent role in stakeholder-oriented communication, they are often seen as ambassadors for the company within the company [59,61]. Based on theoretical and logical considerations, H3 is not rejected, but the identified limitation must be considered in the interpretation.

H4 suggested that KO influences WB. The results obtained (β = 0.679; t-value = 12.525; *p* < 0.05; f^2^ = 0.856) indicate a high intensity. Previous studies have shown that an attractive working environment and a positive working atmosphere through interlinked communication, organizational, and training concepts increase employee productivity [62,63]. In addition, a modern corporate culture helps to create a productive and positive working and learning atmosphere [68]. Therefore, H4 is confirmed.

H5 suggested that WB influences AR. The results obtained (β = 0.326; t-value = 2.566; *p* < 0.05; f^2^ = 0.088) indicate that the intensity is moderate. Previous studies have shown that structured training is a critical success factor [88]. The employer image of companies that invest in the further training of their employees is generally perceived positively [65,66]. Therefore, H5 is confirmed.

H2 and H4 were the two hypotheses with the strongest positive results. They relate to further training perspectives and the work atmosphere and more exactly to the communication around these topics. This means that the signaling theory must be used to generate employee satisfaction regarding the work atmosphere and further training opportunities. This is a continuous action that needs to be considered in the HR strategies of the companies in Germany. These two aspects could even be put forward and advertised when recruiting new talents and their performance tested against the retention quotas of employees. By putting forward a positive work atmosphere and the opportunities for further training in the job descriptions, companies that hire in tense sectors might benefit from more job applications and then from higher employee retention. Regarding the work atmosphere, employees place a high value on good conflict resolution strategies, on effective communication, and on high trust between colleagues and management. When it comes to further training, employees want further training opportunities to reach them in a timely and open manner, to receive training they can put into practice in their daily office life, and to advance their careers within a company thanks to further training.

## 6. Conclusions

### 6.1. Theoretical and Management Implications

In the digital age and in the context of the ongoing shortage of skilled workers, organizational strategies for employer branding must be continuously evaluated, as must employee satisfaction. The aim of this study was to determine the status quo of the communication culture of the organization under investigation and to use the findings as a basis for the development of an employer branding strategy based on continuous improvement of the employer image. The data collected for the company shows the relevance of an open communication culture in the interplay among the working atmosphere, further training, and employer image. To compete successfully for qualified specialists and talent, companies in the social economy must strive to continuously improve their employer image. The results of this study show that the employer image can be improved through the successful use of signaling theory approaches in hiring, organizational commitment, and holistic qualification through internal and/or external training measures for employees. When companies in this sector enter the labor market, they are often accompanied by a negative image. Many skilled workers relevant to these companies are conditioned by negative media reports so the intention to become professionally involved in this sector is often severely limited. To counteract the shortage of skilled workers, companies in the social economy are forced to position themselves as attractive employers in the labor market. To do this successfully, companies must continuously improve their image as employers and communicate this to the external and internal labor market. This is a top-down process and requires active support from management so that the measures developed take effect at all organizational levels. This is the only way to achieve the goal of recruiting suitable specialists and retaining them in the long term. Achieving this goal makes an important contribution to securing the company’s success. In practice, it is therefore particularly important to determine the company’s individual status quo and to research the relevant factors of employer image as perceived by the target group. This mindset is the first step towards successfully building a positive employer image. Hypotheses 2 and 4 showed the strongest positive influence when it came to the role of communication culture in the interaction with the working atmosphere, further training, and employer image. This suggests that a positive work atmosphere has an overall positive influence on communication in the organization. Furthermore, the further development of internal stakeholders is actively promoted, which leads to a strategic competitive advantage. In addition, it was found that structured training and development initiatives improve the perception of the social economic organization. Building on this, an employer brand can then be developed that communicates the company’s positive image both internally and externally. In addition, employees who identify with the company and can act as ambassadors for the company are needed. The first step should therefore be to continue to inspire and retain existing employees. A social economy organization that establishes an honest communication culture, shares information openly, and promotes employee development can continuously improve its perceived employer image. The signals used in communication to employees stimulate expectations that management’s actual actions must match as closely as possible. If this is not adhered to, the psychological contract patterns will be violated, which will have psychosocial effects on the affected employees and ultimately also on the SEO’s attractiveness as an employer.

### 6.2. Recommendations for Further Research

The present study has limitations and thus provides potential for further empirical projects. The sample consists of employees from one SEO in Germany. One of the main limitations of this study is the sample size and the fact that this study was limited to Germany. This study could be replicated with a larger sample in Germany. It would also be interesting to replicate this study in other countries in the EU and even worldwide and then compare the results obtained. Further studies can transfer the research model used to other SEOs. The study design can also form the basis for a comprehensive industry study. Supplementary research activities can take other dimensions into account to expand the hypothesis system and draw new conclusions. Furthermore, the effect of other variables (items from the questionnaire) can be analyzed. The focus of this sector-specific study was on employer image. Therefore, future research activities will focus on the central role of training and further education as well as organizational mindset in achieving the formulated employer branding goals. A further need for research arises from the psychological contracts that arise tacitly in companies. Their relevance for the relationship between employee and employer, for the employer image, and the credible communication of the employer brand is an element that receives little attention in many companies. Empirical research into the psychosocial effects of compliance with and violation of psychological contract patterns and their use as a personnel policy instrument has received little attention in business administration. Communication (of mutual expectations) is also a key factor in successfully shaping the working relationship.

## Figures and Tables

**Table 1:** Compressed overview of the MAB projects at the main site. Source: Own representation.

MAB Project 2020	MAB Project 2023
Questionnaires issued n = 94 RLQ = 70% (n = 66) Employee satisfaction index: ESI ± SD = 2.57 ± 1.06	Questionnaires issued n = 129 RLQ = 75% (n = 97) Employee satisfaction index: ESI ± SD = 2.43 ± 1.04

**Table 2:** Correlations between the constructs. Source: Own representation.

		Gender						
		Male		Female		No Information		∑
		n	%	n	%	n	%	
Age	<30 years	15	51.7	14	48.3	0	0.0	29
	30–50 years	19	42.2	25	55.6	1	2.2	45
	>50 years	6	46.2	7	53.8	0	0.0	13
	No information	0	0.0	1	10.0	9	90.0	10
Company affiliation	<3 years	15	41.7	21	58.3	0	0.0	36
	3–10 years	17	43.6	21	53.8	1	2.6	39
	>10 years	8	66.7	4	33.3	0	0.0	12
	No Information	0	0.0	1	10.0	9	90.0	10

**Table 3:** Correlations between the constructs. Source: Own representation.

	Construct	1	2	3	4
1	Working atmosphere	1.000			
2	Employer image	0.527	1.000		
3	Communication	0.531	0.556	1.000	
4	Further training	0.584	0.602	0.679	1.000

**Table 4:** Criteria for assessing the quality of the measurement model. Source: Own illustration based on [81,84].

Quality Criterion	Type of Quality	Quality Measure	Demand Level
Content validity		Conceptualization and operationalization based on the theoretical frame of reference	Qualitative preliminary phase, pretest, qualitative analysis
Reliability			
		Indicator relevance	Qualitative analysis
	Weights	Significance of the indicator weights (t-value)	t ≥ 1.960 (5% probability of error)
	Multicollinearity	Variance inflation factor (VIF)	VIF ≤ 10

**Table 5:** Statistical characteristics of the quality measures of the formative measurement model. Source: Own representation.

Construct	Description	Formative Indicators	SD	Weight	Load	VIF
Communication KO adapted after [79,85]	I receive timely and sufficient information that I need for my area of work.	KO01	0.854	0.163	0.711	1.919
	The exchange of information among the departments/staff units works very well.	KO02	0.845	0.186	0.713	1.802
	The activities of the individual employees are coordinated.	KO03	0.726	0.282	0.773	1.836
	Suggestions for improvement and tips are taken seriously, discussed, and implemented.	KO04	0.954	0.599	0.892	1.419
Working climate BK adapted after [79,85]	We trust each other so much that we can talk openly about everything.	BK01	1.082	0.370	0.845	1.804
	There is mutual support and assistance among my colleagues.	BK02	0.950	−0.039	0.480	1.368
	Conflicts are dealt with openly and resolved objectively.	BK03	1.126	0.734	0.961	2.112
Continuing education WB	I receive sufficient information about further education and training opportunities.	WB01	1.158	0.232	0.736	1.554
	I can put into practice what I have learned in the training courses and at the IBF.	WB02	0.895	0.343	0.800	1.589
	I am able to realize my professional goals in this company.	WB03	1.094	0.600	0.925	1.739
Employer image AR	As an employee, I see that the company’s image on the market is excellent.	AR01	0.724	0.462	0.797	1.308
	I can recommend this company as an employer to friends, acquaintances, or colleagues.	AR02	0.659	0.691	0.915	2.112

Note: load > 0.70; SD-Standard Deviation; VIF-Variance Inflation Factor < 5.

**Table 6:** Criteria for assessing the quality of the structural model. Source: Own illustration based on [82].

Type of Quality	Quality Criterion	Demand Level
Testing the constructs	Coefficient of determination (R^2^)	R^2^ ≥ 0.667 (substantial) R^2^ ≥ 0.333 (moderate) R^2^ ≥ 0.199 (weak)
Checking the path coefficients	Standardized path coefficients (β)	≥+0.1 (with positive correlation) ≤−0.1 (with negative correlation)
	t-value	t ≥ 1.960 (5% probability of error)
	Effect size (f^2^)	f^2^ ≥ 0.350 (substantial) f^2^ ≥ 0.150 (moderate) f^2^ ≥ 0.020 (weak)

**Table 7:** Statistical values of the coefficients of determination R^2^. Source: Own representation.

Construct	Coding	R^2^
Working atmosphere	BK	0.282
Employer image	AR	0.434
Communication	KO	•
Further training	WB	0.461

**Table 8:** Testing the hypotheses. Source: Own representation.

Hypothesis (H)	Path	Coding	Presumed Influence	Path Coefficient	SD	t-Value	*p*-Value	95% Confidence Interval	VIF	f^2^	H Accepted?
H1	Working atmosphere → Employer image	BK → AR	+	0.222	0.135	1.647	0.100	[0.034; 0.498]	1.599	0.054	✔ *
H2	Communication → Working atmosphere	KO → BK	+	0.531	0.071	7.506	0.000 *	[0.022; 0.456]	1.000	0.394	✔
H3	Communication → Employer image	KO → AR	+	0.217	0.122	1.780	0.075	[0.408; 0.682]	1.957	0.042	✔ *
H4	Communication → Further training	KO → WB	+	0.679	0.054	12.525	0.000 *	[0.572; 0.790]	1.000	0.856	✔
H5	Further training → Employer image	WB → AR	+	0.326	0.127	2.566	0.010 *	[0.065; 0.558]	2.129	0.088	✔

Note: * *p* < 0.05; ✔ * = hypothesis partially accepted; ✔ = hypothesis confirmed.

## Data Availability

Data will be made available upon request from the corresponding author.
